# A Method to Determine Lysine Acetylation Stoichiometries

**DOI:** 10.1155/2014/730725

**Published:** 2014-07-20

**Authors:** Ernesto S. Nakayasu, Si Wu, Michael A. Sydor, Anil K. Shukla, Karl K. Weitz, Ronald J. Moore, Kim K. Hixson, Jong-Seo Kim, Vladislav A. Petyuk, Matthew E. Monroe, Ljiljiana Pasa-Tolic, Wei-Jun Qian, Richard D. Smith, Joshua N. Adkins, Charles Ansong

**Affiliations:** ^1^Biological Science Division and Environmental, Pacific Northwest National Laboratory, Richland, WA 99352, USA; ^2^Bindley Bioscience Center, Discovery Park, Purdue University, West Lafayette, IN 47907, USA; ^3^Molecular Sciences Laboratory, Pacific Northwest National Laboratory, Richland, WA 99352, USA; ^4^Institute for Basic Science, Seoul National University, Seoul 151-747, Republic of Korea

## Abstract

Lysine acetylation is a common protein posttranslational modification that regulates a variety of biological processes. A major bottleneck to fully understanding the functional aspects of lysine acetylation is the difficulty in measuring the proportion of lysine residues that are acetylated. Here we describe a mass spectrometry method using a combination of isotope labeling and detection of a diagnostic fragment ion to determine the stoichiometry of protein lysine acetylation. Using this technique, we determined the modification occupancy for ~750 acetylated peptides from mammalian cell lysates. Furthermore, the acetylation on N-terminal tail of histone H4 was cross-validated by treating cells with sodium butyrate, a potent deacetylase inhibitor, and comparing changes in stoichiometry levels measured by our method with immunoblotting measurements. Of note we observe that acetylation stoichiometry is high in nuclear proteins, but very low in mitochondrial and cytosolic proteins. In summary, our method opens new opportunities to study in detail the relationship of lysine acetylation levels of proteins with their biological functions.

## 1. Introduction

Lysine acetylation (KAc) of proteins is a ubiquitous posttranslational modification (PTM) that controls many cellular processes. The dynamic regulation of KAc by lysine acetyltransferases (KATs) and deacetylases (KDACs) modulates many important cellular functions, such as cell metabolism and gene expression [1, 2]. Recent advances in mass spectrometry combined with immunoaffinity purification are enabling the identification and relative quantification of thousands of acetylation sites in a single experiment [3–5]. These new data have boosted the discovery of regulatory functions of KAc for many proteins, including a variety of metabolic enzymes [2]. Although significant progress has been made, a major remaining hurdle in the field is the determination of acetylation stoichiometry on proteins. The knowledge of KAc stoichiometry is considered essential to better understand the mechanism and impact of this modification on the control protein functions, such as enzymatic activity [2, 6]. Indeed, this problem is not exclusive to KAc as there are almost no systematic determinations of the stoichiometry of PTMs. This has remained a challenge because methods to determine stoichiometry of PTMs are not compatible with enrichment procedures, since both modified and unmodified versions of polypeptides need to be present in the sample. Global studies have been successfully performed to determine the stoichiometries phosphopeptides [7, 8]. More recently, Weinert et al. described a sophisticated method to determine the stoichiometry of acetylated lysine residues [9]. They applied the concept developed by Olsen et al. that in different conditions modified peptides should have their abundance changed inversely proportional to their nonmodified counterpart [8]. Thus, Weinert et al. induced the acetylation of proteins with acetylphosphate and compared the increase in abundance to the acetylated peptides to the decrease in their nonmodified counterparts [9].

**Figure 1:**
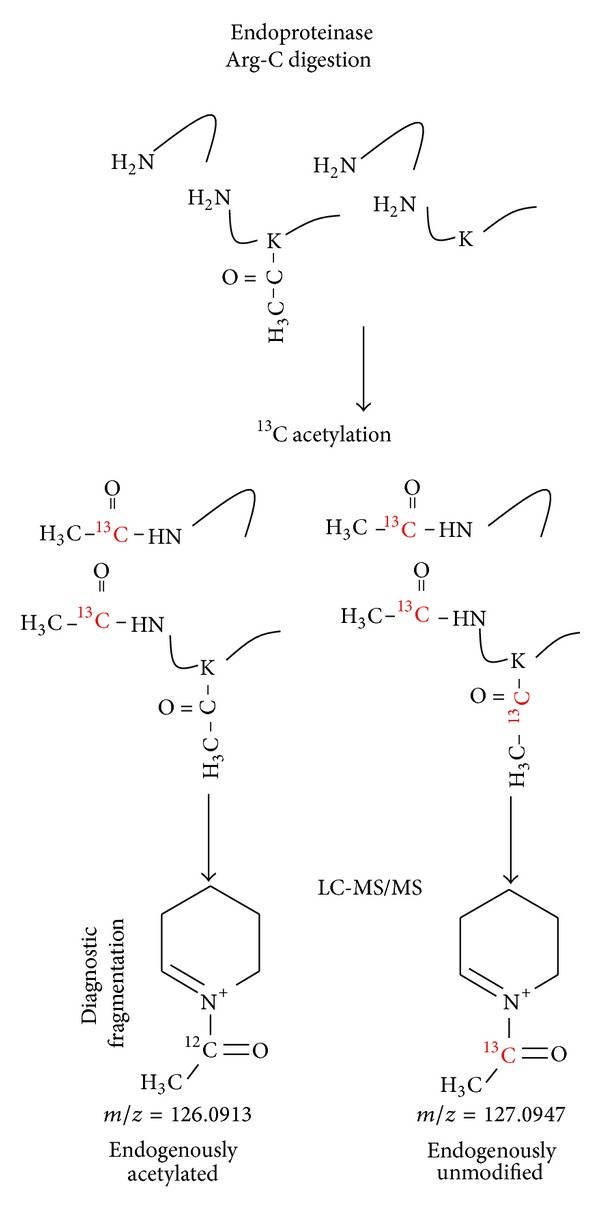
Methodology for determination of lysine acetylation stoichiometry. Proteins are digested with endoproteinase Arg-C, acetylated with 1,1′-^13^C_2_-acetic anhydride, and analyzed by liquid chromatography-tandem mass spectrometry. Upon MS/MS analysis, the fragmentation of acetyllysine generates diagnostic ions, at *m*/*z* 126.0913 (for endogenous acetylated lysine residues) and 127.0947 (for endogenously unmodified lysine residues) that can be used to identify and quantify acetylated peptides.

Herein, we developed an alternative method to determine protein KAc stoichiometries globally based on isotope labeling with the detection and quantification of a small diagnostic fragment ion for KAc found in tandem mass spectrometry (MS/MS) spectra. Small MS/MS fragment ions, such as immonium ions, can be used as unique signatures to detect the presence of specific amino acid residues or posttranslation modifications in peptides. In an effort to identify such diagnostic ions for acetylated lysine residues Kim et al. [10] and Zhang et al. [11] identified a small fragment derived from the fragmentation of acetyllysines at *m*/*z* 126.0913 (Figure 1). Further investigations showed that this diagnostic acetyllysine fragment is indeed highly specific for lysine acetylation and showed that 98.1% of spectra that contained the fragment at *m*/*z* 126.1 were derived from acetylated peptides [12]. Here, we report the use of this acetyllysine diagnostic ion to simultaneously identify and quantify stoichiometries of lysine-acetylated peptides. The method was applied to analyze KAc stoichiometry on proteins from nuclear and whole cell lysates, leading to the identification and quantification of ~750 acetylated peptides. Of note we observe that acetylation stoichiometry is high in nuclear proteins, but very low in mitochondrial and cytosolic proteins, suggesting that most acetylation has a very low stoichiometry in mammalian cells, in agreement with the recent analysis of the stoichiometry of acetylation in the lower eukaryote yeast proteins by Weinert et al. [9]. We also discuss some potential pitfalls of the method and possible applications for studying physiological functions of acetylation. 

## 2. Materials and Methods

### 2.1. Cell Culture and Treatment

RAW 264.7 cells (American Type Culture Collection, ATCC) were cultivated in 150 mm plates at 37°C and 5% CO_2_ atmosphere in 20 mL DMEM (Cellgro) supplemented with 10% fetal bovine serum (FBS), 100 U/mL penicillin, and 100 *μ*g/mL streptomycin. When stated, cells were treated for 18 h with 2 mM sodium butyrate diluted in fresh medium. Cells were then washed twice with PBS containing 1 mM sodium butyrate, scrapped out from the plates, and centrifuged at 1000 rpm for 5 min. Cell pellets were submitted to nuclear enrichment or whole cell proteins were extracted prior to the digestion procedure.

### 2.2. Enrichment of Nuclear Fraction

Nuclei were enriched following a previously reported protocol with a few modifications [13]. Cell pellet from the equivalent of one 150 mm plate was resuspended in 1 mL extraction buffer (10 mM HEPES, pH 7.9, 10 mM KCl, 1.5 mM MgCl_2_, 0.34 M sucrose, 10% glycerol, 0.4% triton X-100, 10 mM N-ethylmaleimide (NEM), 2 mM tris(2-carboxyethyl)phosphine (TCEP), 10 mM sodium butyrate, and 1x Halt protease inhibitor cocktail (Thermo Fisher Scientific)) and incubated for 10 min on ice with occasional rotation. The sample was centrifuged at 6,500 ×g for 5 min at 4°C, and the pellet containing nuclei was washed twice by resuspending with 1 mL washing buffer (10 mM HEPES, pH 7.9, 10 mM KCl, 1.5 mM MgCl_2_, 0.34 M sucrose, 10% glycerol, 10 mM NEM, 2 mM TCEP, 10 mM sodium butyrate, and 1x Halt protease inhibitor cocktail) and centrifuging at the same conditions.

### 2.3. Western Blot Analysis

Nuclear extracts were separated in 4–12% NuPAGE (Invitrogen) and transferred onto polyvinylidene fluoride (PVDF) membranes according to the manufacturer recommendations. After blocking the membrane with membrane for 2 h with 5% nonfat milk the membranes were probed with polyclonal anti-H4K12Ac or polyclonal anti-histone H4 (39270, Active Motif) and followed by incubation with secondary horseradish-conjugated anti-rabbit IgG. Blots were developed with ECL reagent (Pierce) and visualized in a FluorChem Q imaging system (Alpha Innotech). Band densitometries were analyzed with GIMP v.2.8 (http://www.gimp.org/).

### 2.4. Protein Digestion

Whole cell pellets and enriched nuclei fractions were extracted with 0.5% sodium dodecyl sulfate and 10 mM sodium butyrate, and the protein content was quantified using a bicinchoninic acid assay (Thermo Fisher Scientific). 250 *μ*g of proteins were precipitated with 3 volumes of cold acetone for 2 h at −20°C and centrifuged at 16,000 ×g for 20 min at 4°C and the pellet was washed with 1 mL cold acetone and dried in the chemical hood. Whole cell lysate, nuclear proteins, and bovine serum albumin (standard) were dissolved in 100 *μ*L 100 mM NH_4_HCO_3_ containing 8 M urea and 5 mM TCEP and incubated for 1 h at 37°C. The reaction was diluted with 100 mM NH_4_HCO_3_ to obtain a final concentration of 2 M urea and CaCl_2_ was added to a final concentration of 1 mM. Proteins were digested with 1/100 (enzyme/protein ratio) endoproteinase Arg-C (Protea Biosciences) overnight at 37°C. After the digestion, the reduced thiol groups were alkylated by adding NEM to a final concentration of 2 mM and incubating for 1 h at 37°C. Digested peptides were desalted using C18 SPE cartridges (50 mg, Discovery, Sulpelco), as previously described [14], and dried in a vacuum centrifuge.

### 2.5. Acetylation of Primary Amines

Digested peptides were resuspended in 100 *μ*L 250 mM triethylammonium bicarbonate, and then 30 *μ*L acetylating reagent (25% acetic anhydride (regular or 1,1′-^13^C_2_-acetic anhydride, both from Sigma-Aldrich) diluted in anhydrous acetonitrile) was added and the pH was adjusted to 8.5 with 2 M NaOH. The reaction was incubated for 1 h at 25°C. To revert possible undesirable* O*-acetylation, 5 *μ*L 50% hydroxylamine solution (Sigma) was added and the samples were incubated for 30 min at room temperature. Peptides were then desalted with C18 SPE cartridges and dried in a vacuum centrifuge. 

### 2.6. Proteomic Analysis

Acetylated peptides derived from the digestion of BSA and nuclear fractions were submitted to LC-MS/MS analysis on a Waters NanoAquity system with a custom packed C18 column (70 cm × 75 *μ*m i.d., Phenomenex Jupiter, 3 *μ*m particle size, 300 Å pore size). The elution was carried out in a flow rate of 0.3 *μ*L/min, by equilibrating with 100% mobile phase A (0.1% formic acid in water) for 2 min, followed by a 200 minute RPLC gradient (0–12% mobile phase B (0.1% formic acid in pure acetonitrile) over 20 min, 12–35% B over 120 min, 35%–95% B over 20 min, and 95% buffer B for 10 min). Eluting peptides were directly analyzed by electrospray ionization (ESI)—mass spectrometer (MS) in a Q-Exactive mass spectrometer (Thermo Fisher Scientific). Full-MS scans were collected in a range of 400–2000 *m*/*z* and the 10 most intense peaks were submitted to HCD fragmentation (3.5 *m*/*z* isolation width; 24% normalized collision energy), before being dynamically excluded for 1 min.

Acetylated peptides derived from the digestion of whole cell lysates were separated into 16 fractions by high pH reverse phase chromatography [15] on an Agilent 1100 series HPLC using a Waters XBridge C18 5 *μ*m 4.6 × 250 mm column. Peptides were eluted with flow rate of 0.5 mL/min and a gradient of increasing organic solvent B′ (10 mM ammonium formate in 90% acetonitrile, pH 10) displacing the aqueous solvent A′ (10 mM ammonium formate, pH 10): 0–5% B′ over 10 min, 5–35% B′ over 60 min, 35–70% B′ over 15 min and held at 70% B′ for another 10 min. Eluting peptides were collected into 96 fractions and then combined into 16 fractions, before reducing their volumes using a vacuum centrifuge. Each fraction was then submitted to LC-MS/MS analysis. The LC system was custom built using two Agilent 1200 nanoflow pumps and one Agilent 1200 cap pump (Agilent Technologies, Santa Clara, CA), various Valco valves (Valco Instruments Co., Houston, TX), and a PAL autosampler (Leap Technologies, Carrboro, NC). Peptides were loaded in C18 trapping columns (4 cm × 100 *μ*m i.d.) and separated in a longer C18 column (35 cm × 75 *μ*m i.d.) in a flow rate of 0.3 *μ*L/min with a gradient profile as follows: equilibration in 5% B solvent, 5–8% B over 2 min, 8–12% B over 18 min, 12–35% B over 50 min, 35–60% min over 27 min, and 60–95% B over 3 min. Eluting peptides were directly analyzed by ESI-MS/MS in a Velos Orbitrap mass spectrometer (Thermo Fisher Scientific). Full-MS scans were collected in a range of 400–2000 *m*/*z* and the 10 most intense peaks were submitted to HCD fragmentation (3.5 *m*/*z* isolation width; 32% normalized collision energy), before being dynamically excluded for 1 min.

MS/MS spectra were converted to peak lists using DeconMSn (version 2.2.2.2, http://omics.pnl.gov/software/DeconMSn.php) [16] using default parameters and database was searched using MSGF+ against murine protein sequences from Uniprot (downloaded on November 28, 2012) and keratin sequences (32,780 total sequences). As searching parameters, precursor ion mass tolerance was 50 ppm, whereas the fragment mass tolerance is automatically optimized by the searching engine. Peptides with endoproteinase Arg-C digestion in at least one of the termini were considered, with 2 missed cleavages allowed. Cysteine alkylation with NEM (+125.0477 Da) and lysine and N-terminus ^13^C acetylation (+43.0139 Da) were searched as static modifications. Endogenous acetylation (−1.0033 Da, note that this mass shift is compared to the static modification) of lysine side chains and peptide N-terminus and methionine oxidation (+15.9949 Da) were set as variable modifications. The maximum number of variable modifications was set to 3. Spectral-peptide matches were first filtered using a cutoff of MSGF probability [17, 18] ≤5.0*e* − 9 and then each peptide were further filter a MSGF probability ≤2.0*e* − 10, which resulted in ~1% false-discovery rate. Acetyllysine reporter ion (126.0913 and 127.0947 for ^12^C and ^13^C acetyllysine fragments, resp.) intensities were extracted with MASIC (MS/MS Automated Selected Ion Chromatogram Generator, version v2.5.3923, http://omics.pnl.gov/software/MASIC.php) [19] and peptides fragmented multiple times were summed together to remove redundancy in quantification and improve signal to noise ratio.

## 3. Results and Discussion

### 3.1. Method Rational and Experimental Design

Small diagnostic MS/MS fragments, such as immonium ions, have been widely used for detecting the presence of certain amino acid residues or posttranslational modifications in peptides [10–12, 20, 21]. Since MS/MS fragmentation of acetylated lysine residues generates a highly specific diagnostic fragment, we aimed to use it as means to quantify the levels of acetylation on proteins. To achieve this goal, proteins were digested with a protease that cleaves at amino acid residues other than lysines (in our case endoproteinase Arg-C), to obtain both acetylated and nonacetylated peptides with the same lengths. Then the free lysine residues are acetylated with acetic anhydride that contains ^13^C (1,1′-^13^C_2_-acetic anhydride), which leads to an increase of 1.0033 Da for the diagnostic ion. Since ^13^C-containing peptides coelute with peptides with natural isotopic distribution, both endogenously acetylated and ^13^C-acetylated peptides can be fragmented together using a slightly wider isolation window (3.5 *m*/*z* compared to 2.0 *m*/*z*) than that for standard analyses. Subsequently, the light version of the diagnostic ion at *m*/*z* 126.0913 is used to detect peptides that were endogenously acetylated in cells, whereas the heavy version at *m*/*z* 127.0947 is used to determine the proportion of endogenously unmodified lysine residues (Figure 1). Note that the use of deuterated acetic anhydride would not be recommended since deuterium atoms are slightly more hydrophobic and induces a shift in the elution times [22] and the quantitative measurements based on MS/MS spectra could be distorted.

### 3.2. Labeling Efficiency

One of the most challenging steps of this protocol is to obtain highly efficient and selective derivatization of the free lysine residues with acetic anhydride. We first attempted to acetylate proteins with ^13^C-labeled acetic anhydride, but it proved challenging for the reaction to reach completion. Furthermore, prolonged reaction times with increased concentrations of reagent led to the degradation of proteins (data not shown). However, the reaction seems to be far more efficient in peptides compared to proteins. A previous publication showed over 90% of acetylation efficiency for lysine residues when labeling peptides using acetic anhydride [23]. In our laboratory, we have been routinely obtaining over 95% acetylation efficiency (Jong-Seo Kim and Wei-Jun Qian, unpublished observations); thus the error due to inefficient labeling is minor. Another potential issue, although it does not interfere in the quantitative measurements, is side reactions producing* O*-acetylation, but we found that this can be readily circumvented by treating the samples with hydroxylamine as described in Section 2.5.

### 3.3. Method Validation

To validate the method, bovine serum albumin (BSA) was digested with endoproteinase Arg-C, acetylated with both light and heavy acetic anhydride, and mixed in several different proportions prior to liquid chromatography (LC)-MS/MS. The stoichiometry measured for BSA peptides was similar to the proportion of light/heavy samples that were mixed, showing an excellent linear range for quantification with a *R* ^2^ = 0.9948 (Table 1 and Supplementary Table 1 in Supplementary Material available online at http://dx.doi.org/10.1155/2014/730725 (data availability: the raw LC-MS/MS data files are available online at http://www.peptideatlas.org/PASS/PASS00479)). The proportion of light isotope present in the 1,1′-^13^C_2_-acetic anhydride was estimated by judging the abundance of the light acetyllysine diagnostic ion present in BSA acetylated with heavy isotopes. Several peptides showed ~0.5% ^12^C carbon (Supplementary Table 1); thus we conservatively considered peptides with stoichiometries below 1% to be false positives due to the reagent containing measureable levels of the light isotope. It is also worth noting that quantification was performed at peptide levels and not at individual sites, since many peptides contained multiple lysine residues; thus the intensity of the diagnostic ions is derived by the combination of different residues. This issue can be further improved by digesting proteins with other proteases or a combination of different proteases to maximize the likelihood of peptides with single lysine residues. Since the quantification is done in the tandem mass spectra, another potential pitfall is the underestimation of the stoichiometry generated by cofragmenting peptides, which is a well-known phenomenon for isobaric tags for relative and absolute quantification (iTRAQ) experiments [24]. Thus, multidimensional separations in subcellular, proteins, or peptide levels would be recommended to diminish this issue. 

**Figure 2:**
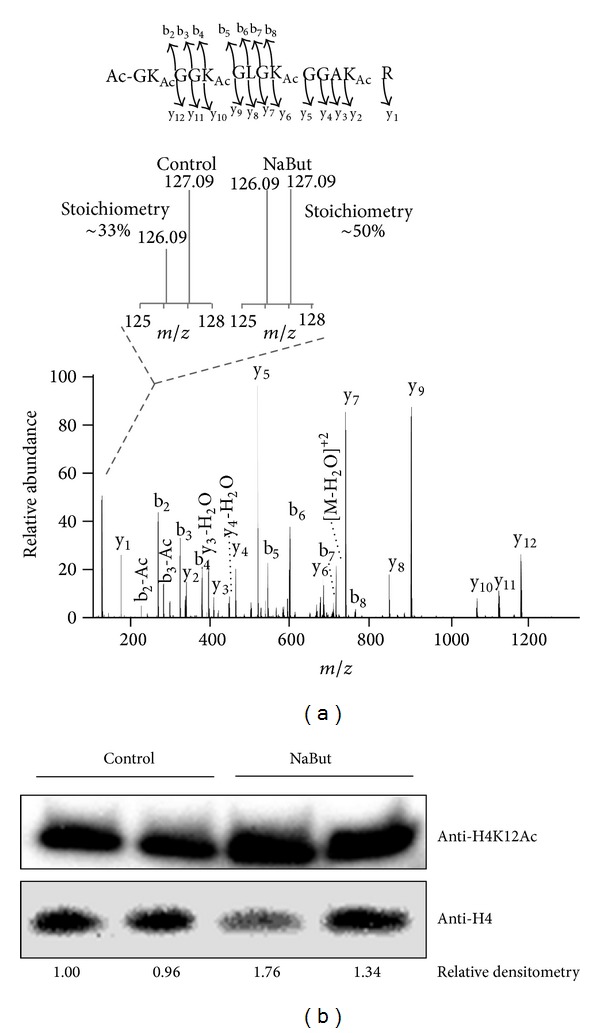
Application of the method to determine lysine stoichiometries for analyzing nuclear proteins. Detection and quantification of lysine acetylation on nuclear proteins from RAW 264.7 cells treated with sodium butyrate (NaBut). (a) An example of identification and quantification of an acetylated peptide derived from histone H4. (b) Western blot against acetylated lysine 12 of histone H4 (H4K12Ac) and total histone H4 (load control). The relative densitometry of each H4K12Ac, normalized by the loading control (histone H4), is shown below each lane.

Having validated the method with an individual protein, we next applied this methodology to study KAc stoichiometries on nuclear proteins. We chose nuclear proteins to further test the method because they comprise several known acetylated proteins and are of moderate complexity compared to whole cell lysates and many of the proteins are well-known to be involved in epigenetic functions. In order not only to determine acetyllysine stoichiometries, but also to detect quantitative differences, RAW 264.7 murine macrophage-like cell lines were treated overnight with 2 mM sodium butyrate (NaBut), a broad spectrum KDAC inhibitor. After the treatment, nuclear fractions were enriched, digested with endoproteinase Arg-C, and acetylated with heavy acetic anhydride prior to reversed phase LC-MS/MS analysis. A total of 486 acetylated peptides were detected and quantified (Supplementary Table 2). Several acetylated peptides were shown to be significantly more abundant in cells treated with NaBut (Supplementary Table 2), consistent with NaBut's role as a broad spectrum KDAC inhibitor. Among these peptides, the N-terminal tail of histone H4 (Figure 2(a)) that included lysine residues K6, K9, K13, and K17 (named in the literature as K5, K8, K12, and K16, resp., due to the processing and removal of the N-terminal methionine residue) exhibited an increase in the relative ratio from ~32% in untreated cells to ~49% after NaBut treatment, which is ~1.5-fold increase in acetylation levels. The levels of acetylation on histone H4 are indeed in a similar range to those previously observed in yeasts [25], even though some differences are expected due to the fact that they are highly divergent species. To cross-validate the results the same samples were submitted to a western blot analysis against the acetylation at K12, which showed a similar increase on acetylation levels (1.34- to 1.76-fold) (Figure 2(b)) after NaBut treatment compared to the mass spectrometry data (1.5- to 1.6-fold) (Supplementary Table 2). Taken together, the results showed that this method enables the detection and quantification of hundreds of acetylated peptides in complex biological samples.

### 3.4. Presence of the Diagnostic Ion

To be suitable for identification and quantification of a PTM, a diagnostic ion needs to be present in the majority of the MS/MS spectra. Thus, the presence of the diagnostic ion for lysine acetylation was investigated in LC-HCD-MS/MS runs derived from the nuclear digests. From the spectra matching lysine-containing peptides 74.3% to 80.0% (77.3% in average) had the diagnostic ion for lysine acetylation (Table 2). This number is similar to that previously described for quadrupole-time-of-flight instruments, which showed the presence of the diagnostic ion for ~70% of the KAc peptides [12]. A detailed analysis of the presence of immonium ions of different amino acid residues in HDC MS/MS spectra showed the presence of these diagnostic fragments to range from 0 to 85% [20]. Thus the presence of the KAc diagnostic ion is in the top of range, making it especially useful for identifying and quantifying KAc peptides.

### 3.5. Application of the Method to Analyze Acetylated Proteins in Whole Cell Lysates

**Figure 3:**
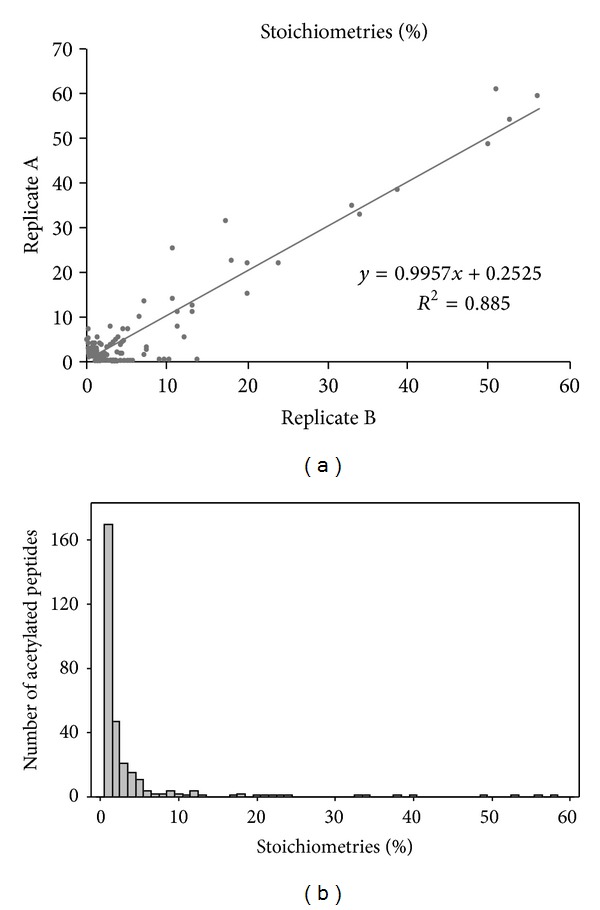
Analysis of lysine acetylation stoichiometries of RAW 264.7 murine macrophage-like cell line. (a) Correlation between the stoichiometries measured in replicates A and B. (b) Distribution of lysine acetylation stoichiometry on proteins from whole RAW 264.7 cell lysates.

We next aimed to determine KAc stoichiometries on proteins from whole cell lysates by analyzing peptides derived from total RAW 264.7 cell extract digestion using two-dimensional LC-MS/MS analysis. A total of 300 acetylated peptides were detected and quantified (Supplementary Table 3); of those 226 were found in both biological replicates and showed a good correlation between them (Figure 3(a)), suggesting a good reproducibility of the method. A distribution of KAc stoichiometries showed that the majority of the peptides had a very low occupancy rate (<10%) for this modification (Figure 3(b)). The low stoichiometry levels may partially reflect the metabolic stage of the cells since KAc is highly dynamic and regulated by the energy availability of the cells [2].

**Figure 4:**
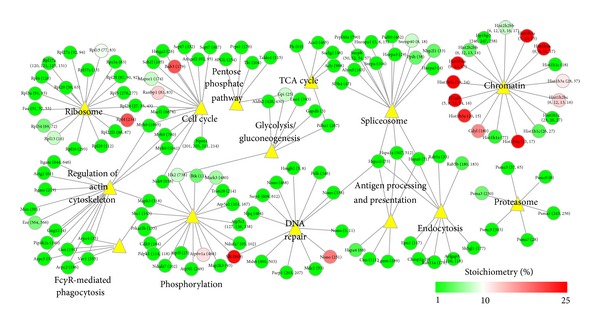
Pathway analysis of lysine acetylation of RAW 264.7 murine macrophage-like cell line. Functions enriched with acetylated proteins. Function-enrichment analysis was performed with Database for Annotation, Visualization and Integrated Discovery (DAVID) and the network built with Cytoscape. Nodes (circles) represent proteins and are connected to their functions (yellow triangles). The node colors represent their acetylation stoichiometries.

To gain more functional insights of different acetylated proteins we performed a function-enrichment analysis, which shows several pathways that are enriched in acetylated proteins, including well-known ones, such as nucleosome [26], regulation of cytoskeleton [27], and central carbon metabolism [2, 28] (tricarboxylic acid cycle, glycolysis/gluconeogenesis, and pentose phosphate pathway) (Figure 4). Furthermore, the network of the enriched functions illustrates that proteins from nucleosome have high stoichiometries, whereas the majority of other functions are rich in proteins with low stoichiometries, at least in our experimental conditions (Figure 4). These observations are in agreement with the recent analysis of the stoichiometry of acetylation in yeast proteins. Weinert et al. found that the acetylation stoichiometry is high in nuclear proteins, while it is low in mitochondrial and cytosolic proteins [9]. Here we show that this phenomenon also applies for higher eukaryotes.

## 4. Conclusions

In summary, we developed a method to determine KAc stoichiometries and tested it for the analysis of single proteins, as well as proteomics studies with samples of medium and high complexities. The application of the method to analyze proteins from a nucleus enriched fraction and whole cell lysates led to the determination of KAc stoichiometry of 743 peptides on 526 proteins. This addresses the challenge of methods to determine stoichiometry of PTMs that are not compatible with enrichment procedures, since both modified and unmodified versions of polypeptides need to be present in the sample. Thus, we believe our method is a timely complement to the recent developments in affinity-based global KAc identification and relative quantification. Furthermore, the combination of our method as a complementary approach with affinity purifications or subcellular fractionations opens new avenues to study in detail the stoichiometry and the dynamics of KAc on individual proteins, protein complexes, cell organelles, and even more complex total cell lysates and tissue samples in the context of gene regulation, cell metabolism, and diseases.

## Supplementary Material

Excel spreadsheets containing peptide identification and stoichiometry details for: validation experiment with BSA, Tab S1: analysis of proteins derived from nuclei of RAW 264.7 cells, Tab S2: and analysis of proteins derived from whole RAW 264.7 cell lysate, Tab S3.

## Figures and Tables

**Table 1:** Acetylation stoichiometry measurements of bovine serum albumin standard.

Measured peptides	Mixed stoichiometry	Measured stoichiometry	Standard deviation
52	0	0.2	0.8
22	1	0.4	0.5
22	10	6.3	3.0
21	50	52.8	9.8
47	100	94.4	2.9

**Table 2:** Presence of the diagnostic ion for lysine acetylation.

Sample	Total PSM∗	Lysine-containing PSM	Spectra containing diagnostic ion *m*/*z* 127.09	Percentage
NaBut-repl.A	6956	4418	3285	74.3%
NaBut-repl.B	4635	3059	2459	80.0%
Control-repl.A	6653	4033	3107	77.0%
Control-repl.B	5533	3559	2819	79.2%

Total	23777	15069	11670	77.4%

*PSM-peptide to spectrum match.

## References

[1] Yang XJ, Seto E (2008). Lysine acetylation: codified crosstalk with other posttranslational modifications. *Molecular Cell*.

[2] Xiong Y, Guan KL (2012). Mechanistic insights into the regulation of metabolic enzymes by acetylation. *Journal of Cell Biology*.

[3] Kim SC, Sprung R, Chen Y (2006). Substrate and functional diversity of lysine acetylation revealed by a proteomics survey. *Molecular Cell*.

[4] Choudhary C, Kumar C, Gnad F (2009). Lysine acetylation targets protein complexes and co-regulates major cellular functions. *Science*.

[5] Lundby A, Lage K, Weinert BT (2012). Proteomic analysis of lysine acetylation sites in rat tissues reveals organ specificity and subcellular patterns. *Cell Reports*.

[6] Rardin MJ, Newman JC, Held JM (2013). Label-free quantitative proteomics of the lysine acetylome in mitochondria identifies substrates of SIRT3 in metabolic pathways. *Proceedings of the National Academy of Sciences of the United States of America*.

[7] Wu R, Haas W, Dephoure N (2011). A large-scale method to measure absolute protein phosphorylation stoichiometries. *Nature Methods*.

[8] Olsen JV, Vermeulen M, Santamaria A (2010). Quantitative phosphoproteomics reveals widespread full phosphorylation site occupancy during mitosis. *Science Signaling*.

[9] Weinert BT, Iesmantavicius V, Moustafa T (2014). Acetylation dynamics and stoichiometry in *Saccharomyces cerevisiae*. *Molecular Systems Biology*.

[10] Kim JY, Kim KW, Kwon HJ (2002). Probing lysine acetylation with a modification-specific marker ion using high-performance liquid chromatography/electrospray-mass spectrometry with collision-induced dissociation. *Analytical Chemistry*.

[11] Zhang K, Tang H, Huang L (2002). Identification of acetylation and methylation sites of histone H3 from chicken erythrocytes by high-accuracy matrix-assisted laser desorption ionization-time-of-flight, matrix-assisted laser desorption ionization-postsource decay, and nanoelectrospray ionization tandem mass spectrometry. *Analytical Biochemistry*.

[12] Trelle MB, Jensen ON (2008). Utility of immonium ions for assignment of epsilon-N-acetyllysine-containing peptides by tandem mass spectrometry. *Analytical Chemistry*.

[13] Shechter D, Dormann HL, Allis CD (2007). Extraction, purification and analysis of histones. *Nature Protocols*.

[14] Ansong C, Yoon H, Porwollik S (2009). Global systems-level analysis of Hfq and SmpB deletion mutants in Salmonella: implications for virulence and global protein translation. *PLoS ONE*.

[15] Wang Y, Yang F, Gritsenko MA (2011). Reversed-phase chromatography with multiple fraction concatenation strategy for proteome profiling of human MCF10A cells. *Proteomics*.

[16] Mayampurath AM, Jaitly N, Purvine SO (2008). DeconMSn: a software tool for accurate parent ion monoisotopic mass determination for tandem mass spectra. *Bioinformatics*.

[17] Kim S, Mischerikow N, Bandeira N (2010). The generating function of CID, ETD, and CID/ETD pairs of tandem mass spectra: applications to database search. *Molecular & Cellular Proteomics*.

[18] Kim S, Gupta N, Pevzner PA (2008). Spectral probabilities and generating functions of tandem mass spectra: a strike against decoy databases. *Journal of Proteome Research*.

[19] Monroe ME, Shaw JL, Daly DS, Adkins JN, Smith RD (2008). MASIC: a software program for fast quantitation and flexible visualization of chromatographic profiles from detected LC-MS(/MS) features. *Computational Biology and Chemistry*.

[20] Michalski A, Neuhauser N, Cox J (2012). A systematic investigation into the nature of tryptic HCD spectra. *Journal of Proteome Research*.

[21] Papayannopoulos IA (1995). The interpretation of collision-induced dissociation tandem mass-spectra of peptides. *Mass Spectrometry Reviews*.

[22] Faca V, Coram M, Phanstiel D (2006). Quantitative analysis of acrylamide labeled serum proteins by LC-MS/MS. *Journal of Proteome Research*.

[23] Zhang Q, Qian WJ, Knyushko TV (2007). A method for selective enrichment and analysis of nitrotyrosine-containing peptides in complex proteome samples. *Journal of Proteome Research*.

[24] Ow SY, Salim M, Noirel J (2009). iTRAQ underestimation in simple and complex mixtures: the good, the bad and the ugly. *Journal of Proteome Research*.

[25] Smith CM, Gafken PR, Zhang Z (2003). Mass spectrometric quantification of acetylation at specific lysines within the amino-terminal tail of histone H4. *Analytical Biochemistry*.

[26] Zentner GE, Henikoff S (2013). Regulation of nucleosome dynamics by histone modifications. *Nature Structural & Molecular Biology*.

[27] Gao YS, Hubbert CC, Lu J (2007). Histone deacetylase 6 regulates growth factor-induced actin remodeling and endocytosis. *Molecular and Cellular Biology*.

[28] Newman JC, He W, Verdin E (2012). Mitochondrial protein acylation and intermediary metabolism: regulation by sirtuins and implications for metabolic disease. *The Journal of Biological Chemistry*.

